# Enhanced Cytotoxic Effects of Arenite in Combination with Active Bufadienolide Compounds against Human Glioblastoma Cell Line U-87

**DOI:** 10.3390/molecules27196577

**Published:** 2022-10-04

**Authors:** Bo Yuan, Jingmei Li, Shin-Ich Miyashita, Hidetomo Kikuchi, Meiyan Xuan, Hirokazu Matsuzaki, Naohiro Iwata, Shinya Kamiuchi, Katsuyoshi Sunaga, Takeshi Sakamoto, Yasuhide Hibino, Mari Okazaki

**Affiliations:** 1 Laboratory of Pharmacology, Graduate School of Pharmaceutical Sciences, Josai University, Keyakidai, Sakado 350-0295, Saitama, Japan; 2 Laboratory of Immunobiochemistry, Graduate School of Pharmaceutical Sciences, Josai University, Keyakidai, Sakado 350-0295, Saitama, Japan; 3 National Institute of Advanced Industrial Science and Technology (AIST), AIST Tsukuba Central 3, 1-1-1 Umezono, Tsukuba 305-8563, Ibaraki, Japan; 4 Laboratory of Pharmacotherapy, Graduate School of Pharmaceutical Sciences, Josai University, Keyakidai, Sakado 350-0295, Saitama, Japan; 5 Laboratory of Organic and Medicinal Chemistry; Graduate School of Pharmaceutical Sciences, Josai University, Keyakidai, Sakado 350-0295, Saitama, Japan

**Keywords:** arsenite, arenobufagin, gamabufotalin, glioblastoma, DNA damage, cell death, cell cycle arrest, combination therapy

## Abstract

The cytotoxicity of a trivalent arsenic derivative (arsenite, As^III^) combined with arenobufagin or gamabufotalin was evaluated in human U-87 glioblastoma cells. Synergistic cytotoxicity with upregulated intracellular arsenic levels was observed, when treated with As^III^ combined with arenobufagin instead of gamabufotalin. Apoptosis and the activation of caspase-9/-8/-3 were induced by As^III^ and further strengthened by arenobufagin. The magnitude of increase in the activities of caspase-9/-3 was much greater than that of caspase-8, suggesting that the intrinsic pathway played a much more important role in the apoptosis. An increase in the number of necrotic cells, enhanced LDH leakage, and intensified G_2_/M phase arrest were observed. A remarkable increase in the expression level of γH2AX, a DNA damage marker, was induced by As^III^+arenobufagin. Concomitantly, the activation of autophagy was observed, suggesting that autophagic cell death associated with DNA damage was partially attributed to the cytotoxicity of As^III^+arenobufagin. Suppression of Notch signaling was confirmed in the combined regimen-treated cells, suggesting that inactivation of Jagged1/Notch signaling would probably contribute to the synergistic cytotoxic effect of As^III^+arenobufagin. Given that both As^III^ and arenobufagin are capable of penetrating into the blood–brain barrier, our findings may provide fundamental insight into the clinical application of the combined regimen for glioblastoma.

## 1. Introduction

Glioblastoma, known as one of the most common and lethal primary brain cancers, has been characterized by high angiogenic and infiltrative capacities [1,2]. Despite advances in the understanding of the disease development, progression, and clinical behavior, the 5-year survival rates of treated glioblastoma remains <5% [3], and the median survival time for patients is still less than 1 year [4,5]. Novel therapeutic approaches are, therefore, urgently needed to fight glioblastoma in view of its resistance to conventional therapies.

Trivalent arsenic derivatives (arsenite, As^III^) such as arsenic trioxide (As_2_O_3_) have shown superior therapeutic efficacy in the treatment of relapsed and refractory acute promyelocytic leukemia (APL) patients [6,7,8]. The successful clinical efficacy in the treatment of APL patients has further opened the possibility of using As^III^ for other malignancies, including solid tumors [8,9]. We have previously performed detailed systematic studies on the metabolites of As^III^ in APL patients [6,7,10], demonstrating for the first time that both inorganic arsenic and methylated metabolites exist in cerebrospinal fluid (CSF), which indicates that As^III^ is capable of penetrating into the blood–brain barrier (BBB) [10]. Additionally, a few previous reports have demonstrated that As^III^ exhibits cytotoxicity against glioblastoma cells by inducing apoptosis, cell cycle arrest, and autophagic cell death [11,12,13]. These previous observations, thus, raise the possibility of repositioning As^III^ to treat glioblastoma patients.

Natural products have been widely reported to potentiate the activity of anticancer drugs such as As_2_O_3_ [14,15]. Bufadienolides are one of the major effective constituents of Huachansu, a well-known Chinese medicine that comes from the dried skin of *Bufo bufo gargarizans* Cantor. Huachansu has been widely used to treat patients with different types of cancers, including hepatoma and lung cancer [16,17]. We have previously shown that active bufadienolide compounds such as gamabufotalin and arenobufagin exhibit selective cytotoxicity toward recalcitrant cancer cells including glioblastoma, with minimal effects on normal human peripheral blood mononuclear cells (hPBMCs) [18] and mouse primary astrocytes [19]. It is worth noting that arenobufagin has been demonstrated to exist in the CSF of arenobufagin-treated rats, directly indicating the capacity of arenobufagin to cross the BBB [19]. In addition, a number of active bufadienolide compounds have been clarified to strengthen the therapeutic efficacy of different types of cancer treatment [20,21]. We also recently demonstrated the enhanced cytotoxic effects of As^III^ in combination with gamabufotalin toward glioblastoma cell lines, which showed much less cytotoxicity to hPBMCs [22]. However, whether arenobufagin possesses the ability to sensitize glioblastoma cells to As^III^ and which mechanisms underlie the action of As^III^ in combination with arenobufagin remain to be seen.

There is little doubt that the cytocidal effect of many anticancer drugs relies on their ability to damage DNA, resulting in an induction of apoptosis/necrosis and cell cycle arrest, consequently inhibiting the proliferation of cancer cells [23,24]. Conventionally, two principal signal pathways of apoptosis have been clarified. The intrinsic mechanism of apoptosis involves a mitochondrial pathway, while the extrinsic pathway is induced by death receptors [8]. Activation of caspases including caspase-9, -8, and -3 plays a crucial role in the initiation and execution of the two signal pathways [8,25]. In addition, activation of the autophagy pathway associated with inactivation of phosphatidylinositol 3-kinase (PI3K)/AKT serine/threonine kinase (Akt) and its downstream mammalian target of rapamycin (mTOR) has been demonstrated in response to DNA damage [26]. A few previous studies have demonstrated that anticancer agents including As^III^ and active bufadienolides induce cytotoxicity in different types of cancer cells, in which inhibition of the PI3K/AKT signaling pathway and cell cycle arrest are involved [19,27,28]. Aberrant activation of Notch signaling has been implicated in maintaining proliferation and survival of human cancer cells, and inhibition of the signaling effectively suppresses tumorigenesis [29,30]. Jagged1, known as an important Notch ligand, is highly expressed in many different types of cancer and has been reported to play a role in promoting cancer progression [30,31]. Emerging evidence has revealed that Jagged1/Notch signaling is highly active and important for initiation and progression in glioblastoma [29,30,31,32]. However, whether the abovementioned cellular processes contribute to the potential cytocidal effect of As^III^ in combination with arenobufagin and how the combined regimen impacts the Jagged1/Notch signaling pathway have not yet been investigated.

In the current study, the cytocidal effect of arenobufagin and gamabufotalin, two active bufadienolide compounds, was first evaluated in a human glioblastoma cell line U-87, aiming to seek a better sensitizer for As^III^. An intracellular arsenic accumulation (As[i]) was also investigated in the cells treated with As^III^ combined with arenobufagin or gamabufotalin. The cytotoxicity of As^III^ in combination with arenobufagin, showing much more efficiency in suppressing U-87 cell growth and upregulating the level of As[i] compared to gamabufotalin, was further investigated by focusing on the induction of apoptosis/necrosis and cell cycle arrest. Caspases activities as well as several vital molecules associated with DNA damage, autophagic cell death, and Notch signaling were also investigated.

## 2. Results

### 2.1. Cytotoxicity Induction in Glioblastoma Cell Line U-87 by As^III^, Arenobufagin and Gamabufotalin

**Figure 1:**
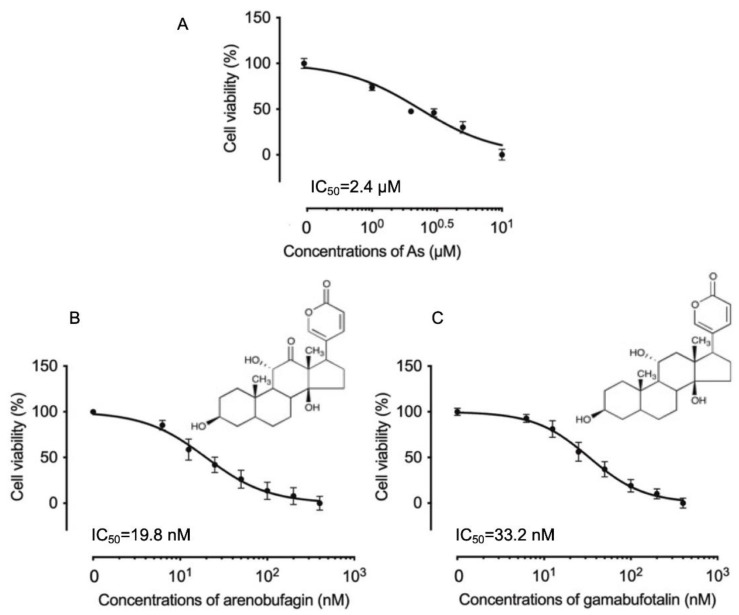
Respective cytotoxic effect of As^III^, arenobufagin, and gamabufotalin against human glioblastoma cell line U-87. Cell viability was determined by CellTiter-Glo Luminescent Cell Viability assay after treatment for 48 h with various concentrations of As^III^ alone (1, 2, 3, 5, and 10 µM) (**A**), arenobufagin alone (**B**), and gamabufotalin alone (**C**) (6.25, 12.5, 25, 50, 100, 200, and 400 nM), respectively. Relative cell viability was calculated as the ratio of the luminescence intensity of each treatment group against those of the corresponding untreated control group. Data are shown as the means ± SD (*n* ≥ 3). As, As^III^.

A significant decrease in cell viability was observed in a dose-dependent manner in U-87 cells, after treatment for 48 h with various concentrations of As^III^, arenobufagin, and gamabufotalin, with each given alone (Figure 1A–C), and the IC_50_ values were 2.4 μM (95% confidence interval, 2.2–2.6; *R*^2^ = 0.9420), 19.8 nM (95% confidence interval, 18.2–21.6; *R*^2^ = 0.9401), and 33.2 nM (95% confidence interval, 30.9–35.7; *R*^2^ = 0.9619) for As^III^, arenobufagin, and gamabufotalin, respectively.

**Figure 2:**
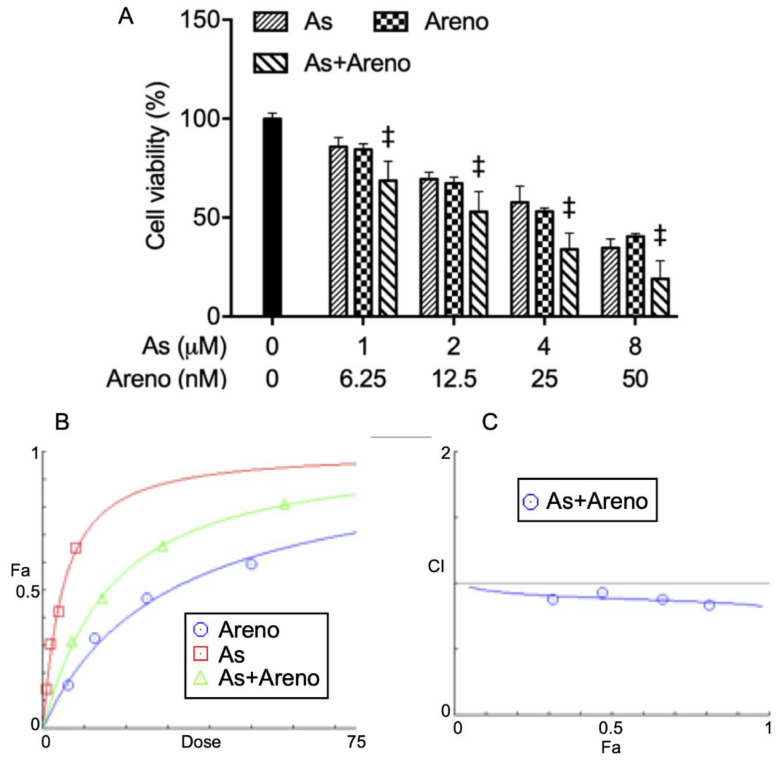
Synergistic cytotoxic effect of As^III^ and arenobufagin in U-87 cells. (**A**) U-87 cells were treated with the combination of As^III^ and arenobufagin at a constant ratio (1 µM As^III^ + 6.25 nM Areno, 2 µM As^III^ + 12.5 nM Areno, 4 µM As^III^ + 25 nM Areno, 8 µM As^III^ + 50 nM Areno). Following treatment for 48 h, cell viability was determined by CellTiter-Glo Luminescent Cell Viability assay. Relative cell viability was calculated as the ratio of the luminescence intensity of each treatment group against those of the corresponding untreated control group. Data are shown as the means ± SD from more than three independent experiments. ‡, *p* < 0.0001 vs. each alone. (**B**,**C**) Combination of As^III^ and arenobufagin exerted synergistic effects on U-87 cells, as reflected by the median-effect method of Chou. The dose-effect curves of single or combined drug treatment analyzed by the median-effect method demonstrated that the values of combination index (*CI*) were < 1, indicating that the two drugs performed in a synergistic manner. As, As^III^; Areno, arenobufagin; Fa, the effect levels; *CI*, combination index.

**Figure 3:**
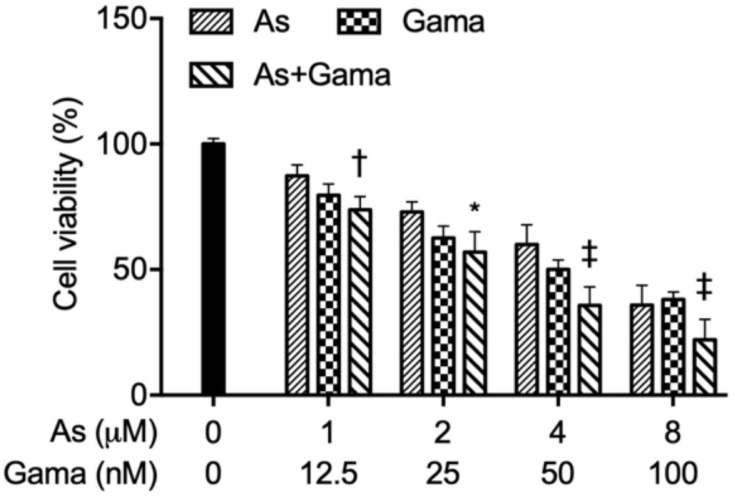
Enhanced cytotoxic effect of As^III^ and gamabufotalin in U-87 cells. U-87 cells were treated with the combination of As^III^ and gamabufotalin at a constant ratio (1 µM As^III^ + 12.5 nM Gama, 2 µM As^III^ + 25 nM Gama, 4 µM As^III^ + 50 nM Gama, 8 µM As^III^ + 100 nM Gama). Following treatment for 48 h, cell viability was determined by CellTiter-Glo Luminescent Cell Viability assay. Relative cell viability was calculated as the ratio of the luminescence intensity of each treatment group against those of the corresponding untreated control group. Data are shown as the means ± SD from more than three independent experiments. *, *p* < 0.05; †, *p* < 0.01; ‡, *p* < 0.0001 vs. each alone. As, As^III^; Gama, gamabufotalin.

To determine if As^III^, in combination with arenobufagin or gamabufotalin, provided generated synergistic, antagonistic, or additive effects against U-87 cells, a two-drug combination at a constant ratio was designed according to the median-effect method of Chou [33,34], based upon the IC_50_ values of each drug. As shown in Figure 2, the combined regimen of As^III^ plus arenobufagin was significantly more cytotoxic than either drug alone (*p* < 0.001; *n* ≥ 3). The values of the combination index (*CI*) were < 1 (Figure 2B,C, Table 1), indicating As^III^ and arenobufagin worked in a synergistic manner in the cells. In the case of the combination of As^III^ and gamabufotalin, although an enhanced cytotoxic effect was induced by the combined regimen compared to either drug alone, synergistic effects were only observed in the cells treated with relatively high concentrations of the two drugs (4 µM As^III^ + 50 nM Gama (*CI* = 0.96042), 8 µM As^III^ + 100 nM Gama (*CI* = 0.98013)) (Figure 3). These results indicated the better effectiveness of the combination of As^III^ and arenobufagin.

### 2.2. As[i] in U-87 Cells Treated with As^III^ in Combination with Arenobufagin or Gamabufotalin

**Figure 4:**
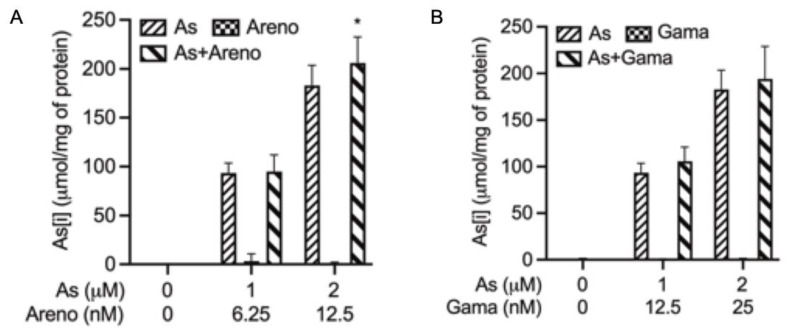
As[i] in U-87 cells treated with As^III^ in combination with arenobufagin or gamabufotalin. Cells were treated for 6 h with As^III^ (1 and 2 μM), arenobufagin (6.25 and 12.5 nM) (**A**), and/or gamabufotalin (12.5 and 25 nM) (**B**), alone and in combination, followed by the assessment of As[i] using ICP-MS, as described in “Materials and Methods”. Data are shown as the means ± SD from more than three independent experiments. *, *p* < 0.05 vs. each alone. As, As^III^, Areno, arenobufagin; Gama, gamabufotalin.

After the exposure of U-87 cells to As^III^, arenobufagin, and/or gamabufotalin, alone and in combination, As[i] was measured by ICP-MS. Since our preliminary experimental data have revealed the largest difference in the level of As[i] between the combined treatment group and single-drug treatment group was at 6 h post-exposure, the following experiments on arsenic accumulation were conducted by exposing the cells to As^III^ (1 and 2 μM), arenobufagin (6.25 and 12.5 nM), and/or gamabufotalin (12.5 and 25 nM), alone and in combination, for 6 h. As shown in Figure 4, the levels of As[i] were approximately two times higher in the cells treated with 2 μM As^III^ than in those treated with 1 μM As^III^ at 6 h post-exposure. It is noteworthy that As[i] was slightly but significantly upregulated in the cells treated with 2 μM As^III^ by the addition of 12.5 nM arenobufaginm in comparison to those treated with As^III^ alone (*p* < 0.05; *n* ≥ 3), although similar upregulation was not observed in the combination of 1 μM As^III^ and 6.25 nM arenobufagin (Figure 4A). In the case of the combination of As^III^ and gamabufotalin (Figure 4B), only a modest increase in As[i] was observed in the U-87 cells treated with As^III^ with the addition of gamabufotalin, in comparison to those treated with As^III^ alone. Moreover, no arsenic accumulation was observed in the cells, regardless of treatment with either arenobufagin or gamabufotalin alone.

### 2.3. Contribution of Apoptosis and Necrosis Induction to the Mode of Action of As^III^ and Arenobufagin, Alone and in Combination, in U-87 Cells

**Figure 5:**
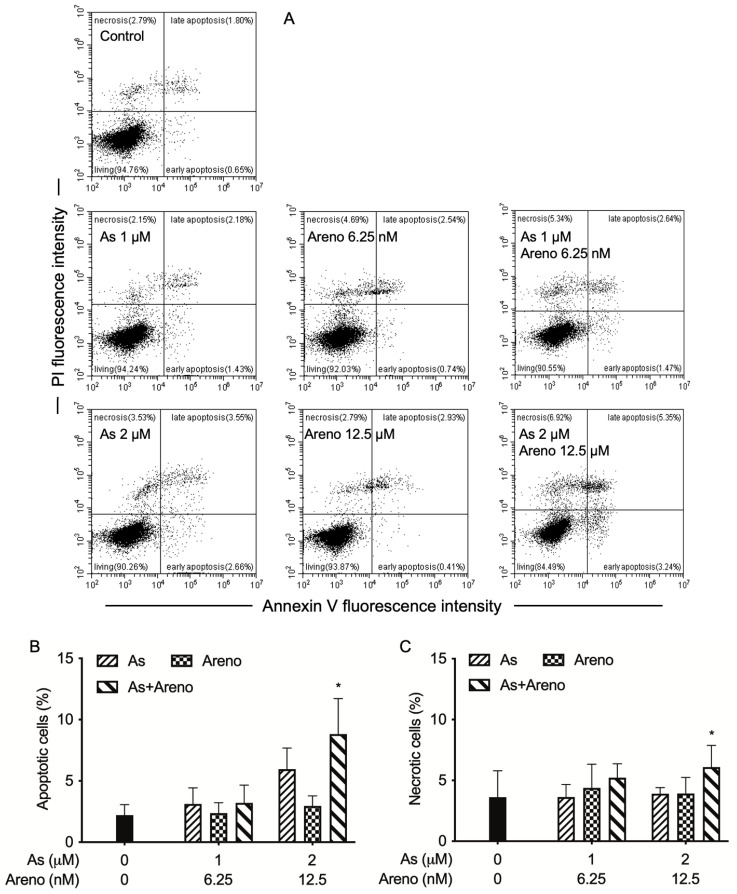
Apoptosis and necrosis induction in U-87 cells treated with As^III^ and arenobufagin, alone and in combination. Following treatment for 48 h with As^III^ (1 and 2 μM) and arenobufagin (6.25 and 12.5 nM), alone and in combination, cells were stained with annexin V-FITC and PI and analyzed by flow cytometry, as described in “Materials and Methods”. Annexin V(−)PI(−) cells, annexin V(+)PI(−)/PI(+) cells, and annexin V(−)PI(+) cells represent viable cells, apoptotic cells, and necrotic cells, respectively. A representative flow cytometry dot plot from three separate experiments is shown (**A**). Quantification in the percentages of apoptotic (**B**) and necrotic cells (**C**) are shown, respectively. Results are shown as the means ± SD from more than three independent experiments. *, *p* < 0.05 vs. each alone. As, As^III^, Areno; arenobufagin.

Since our experimental results have shown the better effectiveness of As^III^ plus arenobufagin, in comparison with gamabufotalin, the mechanisms underlying the synergistic cytotoxic effect of As^III^ and arenobufagin in U-87 cells were further explored in detail. After treatment for 48 h with As^III^ (1 and 2 μM) and arenobufagin (6.25 and 12.5 nM), alone and in combination, annexin V/PI analysis was conducted to explore whether apoptosis and/or necrosis contributed to the cytotoxic effects of As^III^ combined with arenobufagin. As shown in Figure 5, treatment with As^III^ alone caused a dose-dependent apoptosis induction of the U-87 cells, whereas arenobufagin alone did not appear to induce apoptosis in the cells. Of note, in comparison to single-drug treatment, a significant increase in the proportion of apoptotic cells was observed in the cells treated by 2 μM As^III^ plus 12.5 nM arenobufagin (*p* < 0.05; *n* ≥ 3), although only a modest increase was observed in the combined regimen of 1 μM As^III^ and 6.25 nM arenobufagin. Similarly, despite the lack of necrosis-inducing activity of either As^III^ or arenobufagin itself, a modest increase in the proportion of necrotic cells was observed when treated with the combined regimen of 1 μM As^III^ and 6.25 nM arenobufagin, which was significantly strengthened for 2 μM As^III^ combined with 12.5 nM arenobufagin (*p* < 0.05; *n* ≥ 3).

**Figure 6:**
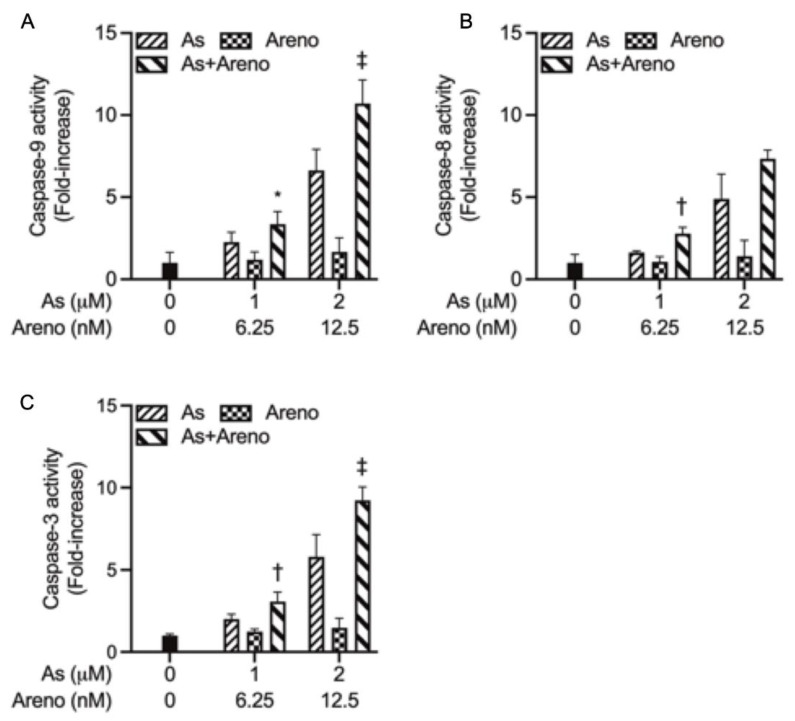
Caspases activation in U-87 cells treated with As^III^ and arenobufagin, alone and in combination. Following treatment with As^III^ (1, 2 µM) and arenobufagin (6.25, 12.5 nM), alone and in combination, the activities of caspase-9 (**A**), -8 (**B**), and -3 (**C**) were measured using a caspase fluorometric assay kit, as described in “Materials and Methods”. Results are shown as the means ± SD from more than three independent experiments. *, *p* < 0.05; †, *p* < 0.01; ‡, *p* < 0.0001 vs. each alone. As, As^III^; Areno; arenobufagin.

To get more detailed information regarding the apoptosis induction, the activation of caspase-9, -8, and -3 was further evaluated in U-87 cells following the treatment for 48 h with As^III^ (1 and 2 μM) and arenobufagin (6.25 and 12.5 nM), alone and in combination. Consistent with Figure 5, treatment with As^III^ increased the activities of caspase-9 (Figure 6A), caspase-8 (Figure 6B), and caspase-3 (Figure 6C) in a dose-dependent manner. Intriguingly, a clear enhancement in their activities was observed by the addition of either 6.25 or 12.5 nM arenobufagin, although arenobufagin itself did not appear to affect caspase activity (Figure 6A–C). It is worth noting that both the combination of 1 μM As^III^ + 6.25 nM arenobufagin (*p* < 0.05 for caspase-9; *p* < 0.01 for caspase-3; *n* ≥ 3) and 2 μM As^III^ + 12.5 nM arenobufagin (*p* < 0.0001; *n* ≥ 3) caused a significant increase in the activities of caspase-9/-3 in U-87 cells (Figure 6A,C); however, a significant increase in the activity of caspase-8 was only observed when treated by 1 μM As^III^ combined with 6.25 nM arenobufagin (*p* < 0.01; *n* ≥ 3) (Figure 6B). Moreover, the magnitude of increase in the activities of caspase-9/-3 was much greater than that of caspase-8 triggered by As^III^ either alone and in combination with arenobufagin, indicating that the activity of caspase-9/-3 was more efficiently intensified by the combined regimen.

### 2.4. Enhanced LDH Release in U-87 Cells Treated with As^III^ in Combination with Arenobufagin

**Figure 7:**
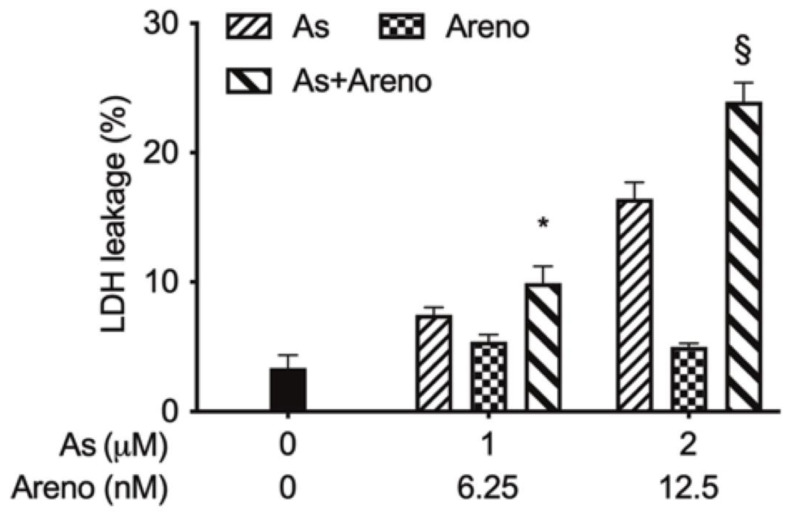
Enhanced LDH release in U-87 cells treated with As^III^ combined with arenobufagin. Following treatment for 48 h with As^III^ (1 and 2 µM) and arenobufagin (6.25 and 12.5 nM), alone and in combination, LDH leakage was measured using the LDH-Cytotoxic test kit, as described in “Materials and Methods”. Results are shown as the means ± SD from more than three independent experiments. *, *p* < 0.05; §, *p* < 0.001 vs. each alone. As, As^III^; Areno; arenobufagin.

The LDH leakage shows clear evidence of cell membrane integrity and cell viability [35,36]. Following treatment for 48 h with As^III^ (1 and 2 µM) and arenobufagin (6.25 and 12.5 nM), alone and in combination, LDH leakage analysis was performed to examine if the treatments impacted cell membrane integrity. As shown in Figure 7, a dose-dependent increase in the LDH leakage was induced by As^III^ alone in U-87 cells. Intriguingly, the addition of arenobufagin prominently enhanced the As^III^-triggered LDH (*p* < 0.05 for 1 μM As^III^ + 6.25 nM arenobufagin; *p* < 0.001 for 2 μM As^III^ + 12.5 nM arenobufagin; *n* ≥ 3), although arenobufagin itself showed little ability to affect LDH leakage.

### 2.5. Induction of G_2_/M Phase Arrest in U-87 Cells Treated with As^III^ in Combination with Arenobufagin

**Figure 8:**
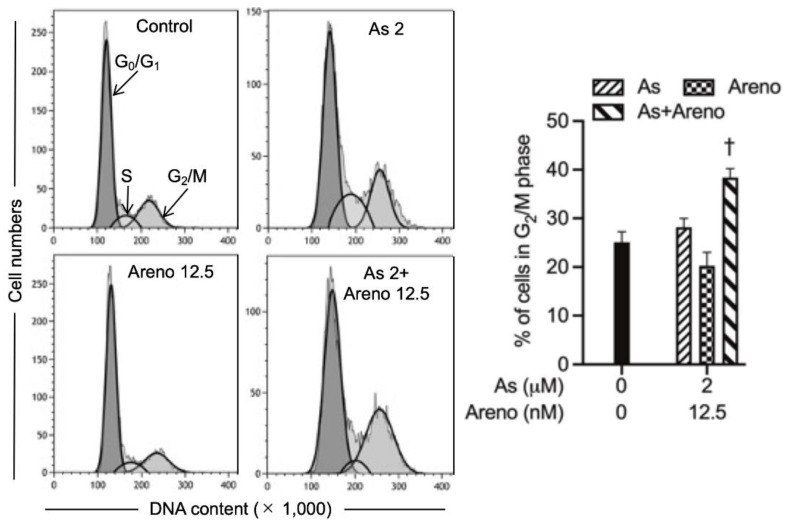
Involvement of G_2_/M arrest in the cytotoxicity of U-87 cells treated with As^III^ combined with arenobufagin. Following treatment with As^III^ (1 and 2 µM) and arenobufagin (6.25 and 12.5 nM), alone and in combination, for 48 h, cell cycle profiling was performed by CytoFLEX S flow cytometer as described in “Materials and Methods”. Results are shown as the means ± SD from more than three independent experiments. †, *p* < 0.01 vs. each alone. As, As^III^; Areno; arenobufagin.

To evaluate if cell cycle arrest is implicated in the cytocidal effects of the combined regimen, cell cycle analyses were performed following the treatment for 48 h with 2 µM As^III^ and 12.5 nM arenobufagin, alone and in combination. As shown in Figure 8, a slight increase in the G_2_/M cell population was induced by 2 μM As^III^ but not by 12.5 nM arenobufagin. Of note, a significant increase in the number of cells in G_2_/M phase was confirmed in the U-87 cells treated with the combined regimen (*p* < 0.01; *n* ≥ 3).

### 2.6. Effect of As^III^ in Combination with Arenobufagin on DNA Damage-, Autophagic Cell Death- and Jagged1/Notch Signaling-Related Gene Protein Expression

**Figure 9:**
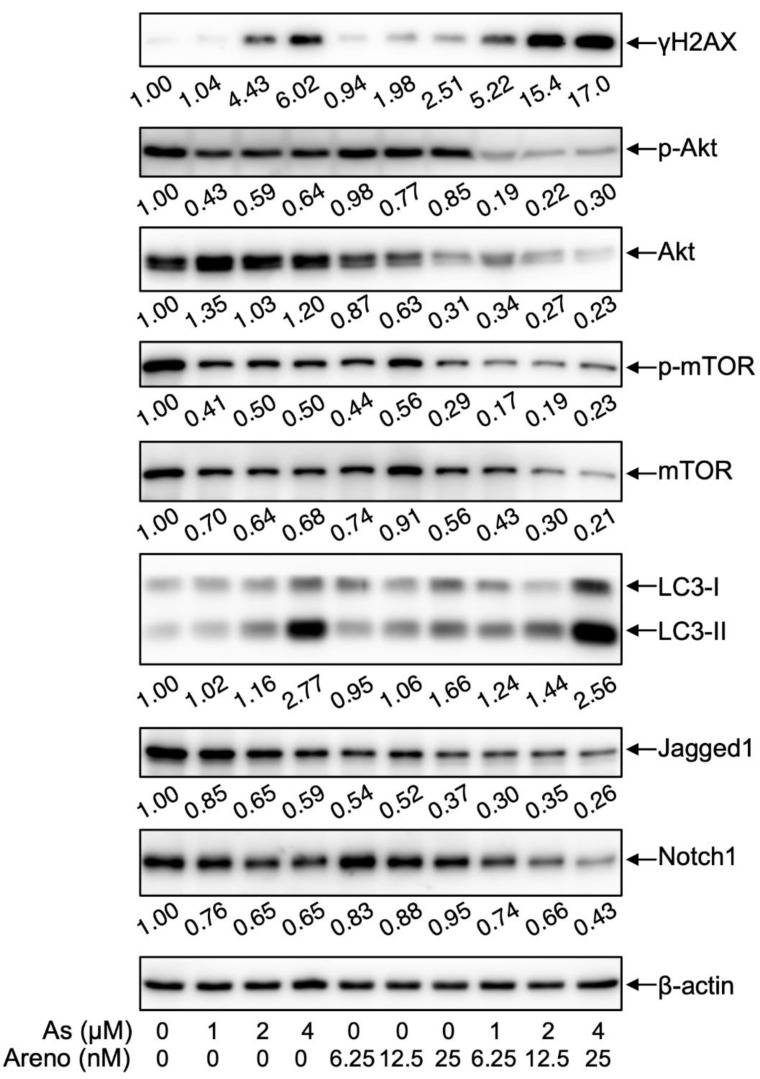
Alteration of DNA damage-, autophagic cell death-, and Jagged1/Notch signaling-related gene protein expression in U-87 cells treated with As^III^ combined with arenobufagin. Following treatment for 48 h with As^III^ (1 and 2 µM) and arenobufagin (6.25 and 12.5 nM), alone and in combination, the expression profiles of each key gene protein were analyzed using Western blotting. A representative image of the expression profile of each protein is shown from three independent experiments. The densitometry of protein bands was analyzed using a program, NIH ImageJ 1.53k. The values under each image represent the ratios between each key molecule and β-actin protein expression levels, which were further compared with those of control group (untreated cells). As, As^III^; Areno, arenobufagin.

It has been demonstrated that As^III^ and arenobufagin induce DNA damage and, consequently, inhibit proliferation of human breast cancer and hepatocellular carcinoma cells, respectively [23,24]. As shown in Figure 9, in line with previous reports, the expression level of γH2AX, a DNA damage marker [23,24], was clearly induced by As^III^ and arenobufagin alone and was further strongly intensified by their combination.

Previous studies have linked the induction of autophagic cell death to the therapeutic effects of various chemotherapeutic agents [14,36,37,38]. In agreement with these previous findings, the expression level of LC3, an autophagic marker [14,22], was obviously upregulated by each single drug and was further dramatically upregulated by their combination. In parallel, the alteration of the expression levels of phosphorylated phospho-mTOR and total-mTOR demonstrated an almost opposite behavior, indicating that the combined regimen not only suppressed activation of mTOR but also reduced its total protein. In addition, a measurable reduction in the expression levels of phosphorylated Akt (phospho-Akt) was induced by both As^III^ and arenobufagin alone. Similar reduction in the expression level of total Akt was also observed in arenobufagin-treated cells, whereas a slight increase in its expression was observed in As^III^-treated cells. Of note, in comparison to each single drug, the combined regimen markedly reduced the expression levels of phospho-Akt and total Akt. These results clearly indicated that the combined regimen caused inhibition of the Akt/mTOR pathway.

Jagged1/Notch signaling maintains proliferation, survival, and angiogenesis in various types of cancer including glioblastoma [29,30,39,40]. In this regard, the expression levels of Notch1 and its ligand Jagged1 were modestly but clearly downregulated in the cells treated with either As^III^ or arenobufagin. Notably, the downregulation was further strengthened by the combination of two drugs, indicating the inhibitory activity of the combined regimen against the Jagged1/Notch signaling pathway.

## 3. Discussion

The results from this study clearly demonstrated that the cytocidal effect of the U-87 cells triggered by As^III^ was significantly intensified by the addition of either arenobufagin or gamabufotalin (Figure 2 and Figure 3). More importantly, synergistic cytotoxic effects were observed in all combined treatment groups following the exposure of U-87 cells to As^III^ in combination with arenobufagin (Figure 2). Interestingly, synergistic effects were only observed in the cells treated with relatively high concentrations of As^III^ and gamabufotalin (4 µM As^III^ + 50 nM Gama, 8 µM As^III^ + 100 nM Gama) (Figure 3), although a previous study demonstrated that As^III^ (1, 2 µM) combined with gamabufotalin ranging from 20 to 50 nM also exhibited synergistic cytotoxicity against U-87 cells [22]. This difference might be attributed to the different cell viability assay system used in each study. In addition, we have previously demonstrated that arenobufagin is able to cross the BBB, as evidenced by the detection of arenobufagin in the CSF of rats that received a single oral dose of the compound [19]. Collectively, we suggest that bufadienolides, especially arenobufagin, may serve as a promising candidate to sensitize glioblastoma cells to As^III^. Of note, Lan et al. have recently demonstrated that active bufadienolides such as gamabufotalin and bufalin exhibit cytotoxicity against glioblastoma cell lines [41,42]. They further suggested that gamabufotalin could be a potent sensitizer of temozolomide, one of the most used clinical drugs for glioblastoma, by triggering a negative feedback loop involving the sodium pump α3 subunit (ATP1A3) and aquaporin 4 to activate p38 MAPK in glioblastoma cells [41]. Additionally, a previous study has demonstrated that downregulation of the expression of sodium pump α1 (ATP1A1) and α3 (ATP1A3) subunits is linked to arenobufagin-triggered cytotoxicity in cervical carcinoma Hela cells [43]. Therefore, investigation into the correlation between the cytotoxicity and the activity of sodium pump and its downstream molecules in U-87 cells treated with As^III^ in combination with arenobufagin obviously needs to be clarified in the future.

Analysis of As[i] further demonstrated that arenobufagin was more efficient than gamabufotalin in upregulating the level of As[i] (Figure 4), reconfirming that the combination of As^III^ and arenobufagin acted effectively to inhibit U-87 cells’ proliferation by manipulating As[i]. It is quite logical to consider As[i] as critical for the modulation of various biological functions and that the levels of As[i] are closely related to arsenic transporters such as P-glycoprotein (P-gp) and multidrug-resistance-associated proteins (MRPs) [8,44]. In this regard, bufalin, another active bufadienolide compound with very similar structure to arenobufagin and gamabufotalin, has been reported to efficiently reverse P-gp-mediated multidrug resistance (MDR) through not only inhibiting the efflux function of P-gp but also downregulating its protein expression in human colorectal cancer cells and it’s xenografts [45]. A previous study has also shown that bufalin can overcome MDR partially through the downregulation of MRP-1 in human hepatocellular carcinoma [46]. Therefore, whether these transporters are involved in the alteration of As[i] warrants further investigation in vitro and in vivo.

From the data, two principal signal pathways of apoptosis, namely the intrinsic and extrinsic pathways, have been identified [8,25]. The intrinsic pathway is mediated by caspase-9, while the extrinsic pathway can be initiated through caspase-8, and the activation of both caspases triggers the execution phase of apoptosis via the activation of the downstream effector caspase, caspase-3 [8,25]. It has been demonstrated that As_2_O_3_ inhibited glioblastoma cell lines, including U-87 via apoptosis induction [11,13]. In line with these previous reports, a dose-dependent induction of apoptosis along with the activation of caspase-9, -8, and -3 was concomitantly observed in U-87 cells treated by As^III^ (Figure 5 and Figure 6). In agreement with our previous report [19], indicating that apoptosis induction was less likely to contribute to the cytotoxicity in U-87 cells caused by arenobufagin, neither apoptotic cells (annexin V-positive cells) nor the activation of caspases was observed in the cells treated with arenobufagin alone (Figure 5 and Figure 6), although arenobufagin has been demonstrated to induce breast cancer MCF-7 cells and cervical carcinoma HeLa cells to undergo apoptosis [43,47], suggesting that induction of apoptosis by arenobufagin may be cell-specific. Moreover, consistent with the synergistic cytotoxic effect of As^III^ and arenobufagin, apoptosis induction associated with the activation of these caspases was further strengthened by the combined regimen of the two drugs in comparison to each single drug (Figure 5 and Figure 6). Of note, the magnitude of increase in the activities of caspase-9/-3 was much greater than that of caspase-8 triggered by As^III^ in combination with arenobufagin (Figure 6A–C), suggesting that the activity of caspase-9/3 was more efficiently intensified by the combined regimen. Our results, thus, demonstrated that both intrinsic and extrinsic pathways were involved in the cytocidal effects of the combined regimen and further suggested that, in comparison to the extrinsic pathway, the intrinsic pathway played a much more important role in the combined regimen-triggered apoptosis. It is worth noting that Bid, a pro-apoptotic Bcl-2 family protein, has been clarified to be responsible for the crosstalk between intrinsic and extrinsic pathways, and that caspase-8-mediated cleavage of Bid into its pro-apoptotic truncated active form uncovers a direct link between the two principal pathways [25,48]. Future studies are needed to address whether there is a crosstalk between the intrinsic and extrinsic pathways, to provide new insight into the cytocidal effects of As^III^ in combination with arenobufagin on U-87 cells.

In addition, agents with the capability to trigger necrotic cell death in a variety of drug-resistant tumor cells have received considerable attention, since defective or inefficient apoptosis is an acquired hallmark of cancer cells [19,36,49]. In this regard, an increase in the number of necrotic cells along with enhanced LDH leakage was concomitantly observed in the cells treated by As^III^ combined with arenobufagin (Figure 5C and Figure 7), suggesting that besides apoptosis induction, necrotic cell death also contributed to the cytotoxic effect of As^III^ and arenobufagin. Similarly, we have recently reported the enhanced LDH release in U-87 cells treated with As^III^ plus gamabufotalin [22]. Our results, thus, suggested that the enhanced cytotoxic effects of As^III^ in combination with active bufadienolide compounds such as arenobufagin and gamabufotalin are partially attributed to the necrosis-inducing activity of the combined regimen. Furthermore, like our previous study showing that the combined regimen of As^III^ plus gamabufotalin triggered G_2_/M arrest in U-87 cells [22], a slight increase in the G_2_/M cell population was induced by As^III^ and further strengthened by the addition of arenobufagin (Figure 8). The capability of arenobufagin to induce G_2_/M phase arrest in a number of cancer cells, including U-87 cells, has been reported [19,23]. Unexpectedly, an obvious arenobufagin-mediated G_2_/M arrest was not observed in our experimental system. It should be noted that Hoechst 33,342 and propidium iodide, two well-known DNA staining reagents, were used to assess cell cycle distributions in the current study and previous studies [19,23], respectively. The discrepancy in the G_2_/M-inducing activity of arenobufagin might be accounted for by the utilization of different DNA staining reagents in different studies. Nevertheless, our findings suggest that G_2_/M arrest is likely to be a general mechanism of As^III^ in combination with active bufadienolide compounds to inhibit cancer cells’ proliferation.

Most chemotherapeutic drugs are considered as DNA-damaging agents with the capacity to induce apoptosis/necrosis and cell cycle arrest and, consequently, inhibit the proliferation of cancer cells [23,24]. Like DNA damage response, autophagy is also one of the biological processes essential for maintaining cellular homeostasis [12,50,51]. Accumulating evidence has demonstrated that autophagic cell death can be activated by DNA damage [26]. PI3K/Akt is usually referred to as a survival mediator involved in cytoprotection [52,53]. Inactivation of PI3K/Akt is conversely associated with the function of mTOR and, thus, positively regulates autophagy [26]. A previous report has demonstrated that arenobufagin induces apoptosis and autophagy via inhibition of the PI3K/Akt/mTOR pathway in human hepatocellular carcinoma cells [53]. In line with these previous reports, the expression level of γH2AX, a DNA damage marker [23,24], was clearly induced by either As^III^ or arenobufagin alone and was further strongly intensified by their combination (Figure 9). Concomitantly, the activation of autophagy, as evidenced by the inactivation of Akt/mTOR and upregulation of LC3 expression, was observed in the cells treated by the combined regimen (Figure 9). Taking the previous results and our observations into account, autophagic cell death associated with DNA damage was partially attributed to the cytotoxicity of As^III^ combined with arenobufagin. We have also recently demonstrated an involvement of similar autophagic cell death in the U-87 cells treated with As^III^ combined with gamabufotalin [22]. In addition, activation of the Notch signaling pathway has been demonstrated to be implicated in the development and progression of various types of cancer including glioma [54]. Among Notch receptors and ligands, Notch1 and its ligand Jagged1 have been found to be associated with the tumorigenesis and recurrence of glioma [54,55]. In the current study, suppression of Notch signaling was confirmed as evidence of the downregulation of the expression levels of Notch1 and Jagged1 in U-87 cells treated with either single drug or their combination (Figure 9). In fact, As_2_O_3_ has been shown to inhibit cell growth and induce apoptosis through the inactivation of Notch signaling pathway in breast cancer cells [39]. Cinobufagin, another active bufadienolide compound, has also been demonstrated to induce the apoptosis of osteosarcoma cells through the inactivation of Notch signaling [56]. Collectively, these results suggested that the inactivation of Jagged1/Notch signaling would probably contribute to the synergistic cytotoxic effect of As^III^ and arenobufagin in U-87 cells.

## 4. Conclusions

Our results demonstrated that both arenobufagin and gamabufotalin sensitized U-87 cells to As^III^-mediated cytotoxicity. We further proposed the better effectiveness of the combination of As^III^ and arenobufagin in the proliferation inhibition of U-87 cells, based on the synergistic cytotoxic effects achieved by As^III^ in combination with arenobufagin, rather than gamabufotalin, in the cells. In support to our proposal, analysis of As[i] also demonstrated that arenobufagin was more efficient than gamabufotalin in upregulating the level of As[i]. In addition to the induction of apoptosis, necrosis, and G_2_/M arrest, our results also suggested that the autophagic cell death associated with DNA damage and the suppression of Jagged1/Notch signaling partially contributed to the synergistic cytotoxicity of the combined regimen. Like arenobufagin, our previous report also suggested the involvement of apoptosis induction in the cytotoxicity of the U-87 cells induced by As^III^ in combination with gamabufotalin [22]. Obviously, further studies on the detailed molecular mechanism of action of As^III^ in combination with arenobufagin and/or gamabufotalin in a mouse xenograft model of human glioblastoma are needed. Given that BBB penetration ability is of critical importance for the efficacy of anticancer drugs against glioma, in vivo antitumor activity of the combined regimen and the concentration profiles of As^III^ and bufadienolides in CSF are warranted.

## 5. Materials and Methods

### 5.1. Materials

Sodium arsenite (NaAsO_2_, As^III^) (>99% purity) and two bufadienolide compounds (arenobufagin and gamabufotalin (≥98% purity)) were purchased from Tri Chemical Laboratories (Yamanashi, Japan) and Baoji Herbest Bio-Tech Co., Ltd. (Baoji, China), respectively. CellTiter-Glo Luminescent Cell Viability Assay kit and Cellstain Hoechst 33342 solution were purchased from Promega Corp. (Madison, WI, USA) and Dojindo (Kumamoto, Japan), respectively. Caspase-9, -8, and -3 Fluorometric Assay Kit was obtained from BioVision (Milpitas, CA, USA). LDH-Cytotoxic Test Wako kit, ClearTrans SP PVDF Membrane (Hydrophobic, 0.2 μm), Protease Inhibitor Mixture, Dulbecco’s Modified Eagle’s Medium (DMEM), and dimethyl sulfoxide (DMSO) were purchased from Wako Pure Chemical Industries (Osaka, Japan). Can Get Signal Immunoreaction Enhancer Solution was purchased from Toyobo CO., LTD. (Osaka, Japan). Fetal bovine serum (FBS) was obtained from Nichirei Biosciences (Tokyo, Japan).

### 5.2. Cell Culture

A human glioblastoma cell line, U-87, was obtained from the American Type Culture Collection (ATCC, Manassas, VA, USA) and cultured in DMEM supplemented with 10% heat-inactivated FBS and antibiotics (100 U/mL of penicillin and 100 μg/mL of streptomycin (Wako Pure Chemical Industries)) in a humidified 5% CO_2_ atmosphere at 37 °C.

### 5.3. Cell Viability Assay

The cytotoxicity of As^III^ and two active bufadienolide compounds (arenobufagin and gamabufotalin) against U-87 cells was measured by CellTiter-Glo Luminescent Cell Viability assay, in accordance with the instructions of the manufacturer. Briefly, the cells were seeded in 96-well plates (Nippon Genetics, Tokyo, Japan) at a density of 1 × 10^4^ cells/well in 0.1 mL medium and cultivated for 24 h. After treatment with the compounds in more than triplicate for 48 h, 100 μL CellTiter-Glo reagent was added into each well, following removal of 100 μL culture medium. The plates were then mixed for 3 min by using an orbital shaker to induce cell lysis. The luminescence intensity was measured with a microplate reader (Wallac. 1420 ARVOsx, PerkinElmer SCIEX, Woodbridge, ON, Canada), following incubation at room temperature for 15 min. The relative cell viability was expressed as the ratio of the luminescence intensity of each treatment group against those of the corresponding untreated control group. Data are shown as mean ± standard deviation (SD) from more than three independent experiments. The IC_50_ value of the drug was calculated using GraphPad Prism9 software. In order to evaluate whether the two drugs generated synergistic, antagonistic, or additive effects, a combination index (CI) was determined, as reported previously, using the computer software ComboSyn (ComboSyn Inc. Paramus, NJ, USA) for drug combinations and for general dose–effect analysis, which was developed by Chou [33,34]. The effect of the combination treatment was defined as a synergistic effect if *CI* < 1, an additive effect if *CI* = 1, or an antagonistic effect if *CI* > 1 [14,36].

### 5.4. Analysis of Arsenic

Cell samples were prepared according to the methods previously described, with slight modifications [7,57]. Briefly, following the exposure of U-87 cells (approximately 4 × 10^5^ cells) for 6 h to relatively low concentrations of As^III^ (1 and 2 μM), arenobufagin (6.25 and 12.5 nM), and/or gamabufotalin (12.5 and 25 nM), alone and in combination, cells were washed three times with cold PBS and harvested in 2% SDS solution. Protein concentrations of the lysate were determined according to Bradford’s method using the protein assay dye reagent (Bio-Rad, Berkeley, CA, USA), in accordance with the instructions of the manufacturer, and using BSA as the standard. Then, a 0.1 mL aliquot of cell lysate was mixed with 0.1 mL nitric acid (HNO_3_; Ultrapur-100, Kanto Chemical, Tokyo, Japan), followed by digestion at 80 °C for 60 min on a dry heat block (EB-303; As One, Osaka, Japan). The samples were diluted 50-fold with deionized water, prepared using a Millipore purification system (Elix; Nihon Millipore Kogyo, Tokyo, Japan), and then analyzed by inductively coupled plasma mass spectrometry (ICP-MS) for total arsenic determination. As[i] was normalized by the amounts of protein and given as micromolar of arsenic per milligram of proteins.

The analysis of total arsenic was performed by ICP-MS according to the methods previously reported, with some modifications [7,57]. The amount of total arsenic in a sample was determined by an internal calibration method, with five points in the 0–10 µg/kg concentration range, using yttrium as an internal standard element. Biological samples, such as blood, contain high concentrations of chloride ion, which interferes with arsenic detection at a mass-to-charge ratio (*m*/*z*) of 75, owing to the formation of argon chloride (^40^Ar^35^Cl) in the argon plasma with the same *m*/*z* as ^75^As. Therefore, the ^40^Ar^35^Cl interference was eliminated by applying the following equation, as recommended in EPA method 200.8 (U.S. EPA. 1994) [58]: 1.000 × ^75^C − 3.127(^77^C − 0.815 × ^82^C), where the C variables represent the calibration blank-subtracted counts for the indicated mass. The analytical conditions of ICP-MS are shown in Table 2.

### 5.5. Annexin V/PI Analysis

TACS Annexin V-FITC apoptosis detection kits (Trevigen, Minneapolis, MN, USA) were used for the detection of early apoptotic and late apoptotic/necrotic cells, according to the method described previously [48,59]. Briefly, following treatment for 48 h with As^III^ (1 and 2 µM) and arenobufagin (6.25 and 12.5 nM), alone and in combination, cells were washed with PBS. Cells (approximately 1 × 10^6^ cells) were then incubated for 15 min in 100 μL of reaction buffer, which contained annexin V-FITC and PI from the kit, followed by addition of 400 μL of binding buffer. Fluorescence intensities of FITC and PI were measured by a CytoFLEX flow cytometer (Beckman Coulter, Brea, CA, USA). A total of 10,000 events were acquired, and data were analyzed by CytExpert Ver 2.4.0.28 software (Beckman Coulter, Brea, CA, USA).

### 5.6. Measurement of Caspases Activity

Activity of caspase-9, -8, and -3 was measured using the caspase fluorometric assay kit, according to the method described previously [59,60]. Briefly, 25 μg/50 μL of protein was plated on a 96-well plate, followed by the addition of 50 μL of 2× reaction buffer, containing 10 mM DTT for each sample, and then 5 μL of 1 mM caspase substrate (final concentration of 50 μM). After incubation at 37 °C for 1 h, the fluorescent intensity (Excitation. 400 nm, Emission. 505 nm) was measured using a microplate reader (SpectraMax Pro M5e, Molecular Devices, San Jose, CA, USA).

### 5.7. Lactate Dehydrogenase (LDH) Assay

After treatment for 48 h with As^III^ (1, 2 µM) and arenobufagin (6.25, 12.5 nM), alone and in combination, LDH leakage from cells (approximately 1–2 × 10^6^ cells) was measured using the LDH-Cytotoxic Test Wako kit according to the method previously described, with slight modifications [14,57]. Briefly, culture supernatants were collected by centrifugation at 2500 rpm for 5 min at 4 °C. Non-treated cells were lysed in culture medium containing 0.2% Tween 20 and mixed aggressively using a vortex mixer, followed by centrifugation at 12,000× *g* for 10 min. Cell lysate was used as the positive control, while culture medium served as the negative control. Culture supernatants were collected and then diluted 16-fold with PBS, and 50 μL of the diluted solution was transferred into wells of a 96-well plate. LDH activities were determined by adding 50 μL of ‘substrate solution’ from the kit, followed by incubation at room temperature for 30 min. The reaction was stopped by the addition of 100 μL of ‘stopping solution’ provided with the kit at room temperature, and the absorbance at 560 nm was measured with a microplate reader (SpectraMax ABS, Molecular Devices, San Jose, CA, USA). Cell damage was calculated as a percentage of LDH leakage from damaged cells using the following formula: LDH leakage (%) = (Sup-NC)/(P-NCT) × 100, where Sup, NC, P, and NCT refer to the absorption of the culture supernatant, negative control, positive control, and culture medium containing 0.2% Tween 20, respectively.

### 5.8. Cell Cycle Analysis

After treatment for 48 h with the indicated concentrations of 2 µM As^III^ and 12.5 nM arenobufagin, alone and in combination, cell cycle analysis was performed using a CytoFLEX S flow cytometer (Beckman Coulter, Brea, CA, USA) according to the methods previously described, with slight modifications [14,36,48]. Briefly, cells (approximately 1 × 10^6^ cells) were washed twice with cold PBS, fixed with 1% paraformaldehyde/PBS on ice for 30 min, washed twice again with cold PBS, permeabilized in 70% (*v*/*v*) cold ethanol, and kept at −20 °C for at least 4 h. Cell pellets were then washed twice with cold PBS after centrifugation (430× *g* for 5 min at 4 °C) and resuspended in 500 µL of Hoechst 33342/PBS (4 µg/mL of Hoechst 33342 in PBS), followed by incubation for 30 min in the dark at room temperature. A total of 10,000 events were acquired for flow-cytometric analysis using CytExpert Ver 2.4.0.28 software. Kaluza Analysis 2.1 software (Beckman Coulter, Brea, CA, USA) was used to calculate the number of cells at G_2_/M phase fraction.

### 5.9. Western Blot Analysis

For preparation of the protein samples, cell pellets (approximately 1–2 × 10^6^ cells per 110 μL buffer) were suspended in Laemmli buffer containing Protease Inhibitor Mixture. Cell suspensions were sonicated (Qsonica, LLC, Newtown, CT, USA) with 10 short bursts of 2 sec followed by intervals of 2 sec for cooling. The suspensions were always kept in an ice bath. Sonicated cells were heated in 95 °C for 5 min and then centrifuged at 13,000× *g* for 15 min at 4 °C. Protein concentrations of the supernatant were determined according to Bradford’s method using the protein dye reagent, in accordance with the instructions of the manufacturer, using BSA as the standard. Western blot analysis was carried out according to a method previously described [14,48]. Briefly, separation of proteins (10–20 μg protein/lane) via sodium dodecyl sulfate polyacrylamide gel electrophoresis was followed by transference to a PVDF membrane, which was then blocked with 5% skim milk/TBST (TBS containing 0.1% Tween-20) for 1 h at room temperature. Protein bands were detected using the following primary antibodies diluted in Can Get Signal Immunoreaction Enhancer Solution: rabbit anti-human β-actin (1:1000 dilution, cat. no. 4967), rabbit anti-human phospho-Histone H2A.X (Ser139) (1:1000 dilution; cat. no. 9718), rabbit anti-human phospho-Akt (Ser473) (1:2000 dilution; cat. no. 4060) and Akt (1:1000 dilution; cat. no. 4691), rabbit anti-human phospho-mTOR (Ser2448) (1:1000 dilution; cat. no. 5536) and mTOR (1:1000 dilution; cat. no. 2938), rabbit anti-human LC3 (1:1000 dilution; cat. no. 12741), rabbit anti-human Notch1 (1:1000 dilution; cat. no. 4380) (Cell Signaling Technology, Danvers, MA, USA), and mouse anti-human Jagged1 (1:500 dilution; cat. no. sc-390177) (Santa Cruz Biotechnology, Inc. Santa Cruz, CA, USA). Blotted protein bands were detected with respective horseradish peroxidase-conjugated secondary antibody and a chemiluminescence (ECL) Prime Western blot analysis system (Amersham Pharmacia Biotech, Buckinghamshire, UK). Relative amounts of the immunoreactive proteins were calculated from the density of the gray level on a digitized image using a program, NIH ImageJ.

### 5.10. Statistical Analysis

Experiments were independently repeated three times, and the results were shown as the means ± standard deviation (SD) of three assays. Statistical analysis was conducted using one-way ANOVA followed by Dunnett’s post hoc test. A probability level of *p* < 0.05 was considered to indicate a statistically significant difference.

## Figures and Tables

**Table 1:** *CI* values of As^III^ at concentrations in combination with Areno in U-87 cells. *CI* < 1 represents synergism. As, As^III^; Areno, arenobufagin; Fa, the effect levels; *CI*, combination index.

As (μM)	Areno (nM)	Fa	*CI* Value
1	6.25	0.314	0.87857
2	12.5	0.472	0.92881
4	25	0.662	0.88209
8	50	0.811	0.83981

**Table 2:** Analytical conditions of ICP-MS.

Instrument	Agilent 7700x ICP-MS(Agilent Technologies, Santa Clara, CA, USA)
RF power	1550 W
Sampling position	10.0 mm
Carrier gas flow rate	1.08 L/min
Monitored ion	As^+^ (*m*/*z* 75), Se^+^ (*m*/*z* 77 and *m*/*z* 82), Y^+^ (*m*/*z* 89)

## Data Availability

The data presented in this study are available on request from the corresponding author.

## Abbreviations

APL: acute promyelocytic leukemia; CSF, cerebrospinal fluid; BBB, blood–brain barrier; mTOR, mammalian target of rapamycin; As[i], intracellular arsenic accumulation; CI, combination index; LDH, lactate dehydrogenase.
